# Live *Malassezia* strains from the mucosa of patients with ulcerative colitis: pathogenic potential and environmental adaptations

**DOI:** 10.1128/mbio.01400-25

**Published:** 2025-06-13

**Authors:** Yong-Joon Cho, Seung Yong Shin, Juan Yang, Hyo Keun Kim, Piyapat Rintarhat, Minji Park, Muhyeon Sung, Kate Lagree, David M. Underhill, Dong-Woo Lee, Sohyeong Choi, Chang Hwan Choi, Chul-Su Yang, Won Hee Jung

**Affiliations:** 1 Department of Molecular Bioscience, Kangwon National University 34962, Chuncheon-si, South Korea; 2 Multidimensional Genomics Research Center, Kangwon National University 34962, Chuncheon-si, South Korea; 3 Department of Internal Medicine, Chung-Ang University College of Medicine, Seoul, South Korea; 4 Department of Systems Biotechnology, Chung-Ang University, Anseong, South Korea; 5 Department of Molecular and Life Science, Hanyang University, Ansan, South Korea; 6 Center for Bionano Intelligence Education and Research, Ansan, South Korea; 7 F. Widjaja Inflammatory Bowel and Immunobiology Research Institute, Cedars-Sinai Medical Center, Los Angeles, California, USA; 8 Division of Immunology, Department of Biomedical Sciences, Cedars-Sinai Medical Center, Los Angeles, California, USA; 9 Department of Pathology and Laboratory Medicine, David Geffen School of Medicine, University of California, Los Angeles, California, USA; 10 Department of Biotechnology, Yonsei University, Seoul, South Korea; 11 Department of Medicinal and Life Science, Hanyang University, Ansan, South Korea; University of Melbourne, Melbourne, Victoria, Australia

**Keywords:** *Malassezia*, inflammatory bowel disease, fungi, gut mucosa, mycobiota

## Abstract

The human gut contains a diverse range of microorganisms, including bacteria, viruses, protozoa, and fungi. Although research has predominantly focused on bacterial populations, emerging evidence highlights the significant role of the gut mycobiota, particularly in the context of inflammatory bowel disease (IBD), including ulcerative colitis (UC) and Crohn’s disease. This study investigates the intestinal mucosal mycobiota of UC patients, aiming to isolate and characterize live *Malassezia* fungal strains from the gut mucosa. Our analysis confirmed the presence of *Malassezia* in the intestinal mucosal layer of UC patients, with live *Malassezia globosa* strains being successfully isolated. As *Malassezia* is generally associated with the skin, the genomic and transcriptomic profiles and virulence of the *M. globosa* gut isolates were compared with those of the skin isolates. While both gut and skin isolates of *M. globosa* shared high genomic similarity, transcriptomic analysis revealed distinct responses to oxygen levels, suggesting niche-specific adaptation. Compared with the skin isolates, the gut isolates exhibited higher virulence in a dextran sulfate sodium-induced mouse colitis model, resulting in more severe disease, reduced survival rates, and elevated proinflammatory cytokine levels in the host. Our findings highlight the potential role of *M. globosa* in the pathogenesis of IBD and underscore the importance of niche-specific adaptations in fungal virulence.

**IMPORTANCE:**

*Malassezia* fungi predominantly reside on human skin and are associated with several skin diseases, such as seborrheic dermatitis. They have also been implicated in various other diseases, including inflammatory bowel disease (IBD). While *Malassezia* DNA has been detected in many fungal microbiome studies using fecal samples, no previous research had isolated live *Malassezia* strains from the gut or confirmed that live *Malassezia* cells reside within the gut environment. In this study, we successfully isolated live *Malassezia globosa* strains from the gut mucosal surface of ulcerative colitis patients and compared them to *M. globosa* skin isolates. Our results revealed significant differences in pathogenicity between the gut and skin isolates and suggest the important role of *M. globosa* in the gut and its involvement in IBD.

## INTRODUCTION

Inflammatory bowel disease (IBD) is a chronic and relapsing-remitting disease that poses a considerable healthcare burden globally. The prevalence of IBD has significantly increased not only in Europe and North America but also in East Asian countries, including Korea. Numerous studies have demonstrated that IBD is caused by various factors, including the genetic susceptibility of the host, dysbiosis of the microbiome, disruption of intestinal barrier functions, and inappropriate immune activation, resulting in the disruption of intestinal homeostasis (1, 2). Ulcerative colitis (UC) and Crohn’s disease (CD) are the two types of IBD. UC occurs as a continuous superficial inflammation mostly in the mucosa and submucosa of the colon, while CD presents as scattered lesions throughout the gastrointestinal tract (3). Western countries, such as those in North America, Western Europe, and Oceania, generally have a higher prevalence of CD than UC (4, 5). Although the total number of IBD cases has increased, the number of UC cases and their annual increase are significantly (approximately more than twofold) higher in Korea (6). The pathogenesis of UC and CD is still not fully understood; however, similar factors influence both types of IBD (7).

The microbiome is a crucial factor in the intestinal environment. In general, the intestinal microbiome in the mammalian host is well controlled by multiple mechanisms, such as the production of antimicrobial peptides and secretion of immunoglobulin A (IgA) and IgG, which can prevent epithelial invasion by microbes. Moreover, mucin, which is a dense hydrogel layer, covers the surface of intestinal epithelial cells and is produced by goblet cells within the epithelium (8). However, numerous studies have reported a shift in the intestinal microbiome in patients with UC and CD (9, 10). Although significant individual variation has been noted, numerous studies have reported common features of alteration of the intestinal microbiome, including reduced bacterial diversity, in patients with IBD. In particular, decreased abundance of Firmicutes phylum and the association of proinflammatory bacteria, such as *Escherichia coli*, *Ruminococcus gnavus*, and *Fusobacterium* species, with IBD have been reported (11).

In addition to the bacterial community, the fungal microbiome (known as the mycobiome) likely plays a crucial role in the gut. Notably, although metagenomic data suggest that fungal DNA accounts for less than 0.1% of the intestinal microbiome, the fungal community must be considered because of the size of fungal cells, which is usually more than 100-fold larger than that of bacterial cells, when studying the intestinal microbiome (12). Accumulating evidence indicates that the mycobiome contributes to the development of IBD, and several fungi commonly observed in multiple studies are associated with individual susceptibility to IBD (12). A pioneering study revealed that the increased abundance of fungal populations, particularly *Candida* species, plays an essential role in antifungal innate immunity in a dextran sodium sulfate (DSS)-induced colitis mouse model (13). *Candida* species are most frequently found in the human intestinal mycobiota and have long been considered to be commensal fungi in the human gut. Although the role of *Candida* species in IBD remains controversial, a clinical study revealed that the abundance of *Candida* species in the intestinal mycobiota of patients with UC is positively correlated with favorable clinical outcomes of fecal transplantation (14). Moreover, numerous studies have demonstrated the augmentation of colitis by oral gavage of *Candida* species in a DSS-induced colitis mouse model (12).

*Malassezia* is considered the predominant fungus in human skin. It is a pathobiont involved in various skin diseases, including psoriasis, seborrheic dermatitis, and atopic dermatitis (15). Studies have indicated a possible role of *Malassezia* species in chronic diseases occurring at other body sites, such as pancreatic cancer and Alzheimer’s disease (16–18). Recent mycobiome analyses have frequently observed *Malassezia* DNA sequence reads in the human gut, indicating that the fungus is one of the core taxa in the human intestinal mycobiota (19–21). A positive correlation between *Malassezia* and *Bacteroides*, which is a predominant bacterial genus in the human gut, has been reported (22). Furthermore, studies utilizing skin swabs and fecal samples from infants suggested that *Malassezia* is transmitted from the mother and colonized in the gut of infants, although the abundance of the fungus gradually decreases upon aging (23, 24). These findings suggest that *Malassezia* resides in both skin and gut mucosa and that the fungus has evolutionarily adapted to a distinct niche-specific environment.

To date, 18 different species have been identified within the *Malassezia* genus (15). Among these, a recent study by Limon et al. using mucosa-washing samples revealed that *M. restricta* and *M. globosa* were strongly associated with CD (25). In that study, a higher abundance of *Malassezia* was noted in the intestinal mucosal surface of patients with CD than in that of healthy individuals. Moreover, in the same study, patients with CD with the IBD risk allele caspase recruitment domain-containing protein (CARD)^S12N^ variant exhibited a tight association with the presence of *Malassezia* in their intestinal mucosa, further confirming the positive correlation between the fungus and the disease (25). CARD9 plays a crucial role in a central signaling pathway in innate immune responses and is activated by pattern recognition receptors, including C-type lectin receptors, such as dectin-1, dectin-2, and mincle, which are key receptors for detecting commensal and pathogenic fungi. Moreover, CARD9^S12N^ is a crucial single-nucleotide polymorphism (SNP) involved in IBD (26). *Malassezia* also exacerbated colitis in a DSS-induced colitis mouse model, which required functional CARD9. Furthermore, dectin-2, but not dectin-1 and mincle, played a crucial role in responding to *Malassezia* in the mouse model (25). Thus, the findings of Limon et al. provide insights into the possible contribution of *Malassezia* to IBD. However, in their study, Limon et al. used *M. restricta* MYA-4611, synonymous with the *M. restricta* type strain CBS 7877, which may not properly represent the fungus within the gut environment because it was originally isolated from the human skin and not from the human gut (27). In general, niche-specific metabolic adaptation, which is normally linked to genomic variations and niche-dependent gene expression, is a well-established phenomenon, particularly in bacteria, based on phenotypic and multiomics analysis (28–30). Fungi also reside in various environments, including different niches within the human host, and multiple selective pressures, such as oxygen depletion, nutrient limitation, and pH fluctuation, lead to niche-specific adaptation (31, 32). To date, no comprehensive study has assessed phenotypic and genetic diversities in the *Malassezia* population in different host niches within the human body to demonstrate niche-specific adaptation of the fungus at the strain level.

In this study, we hypothesized that *Malassezia* adapts to different host niches, such as the skin and gut, and therefore possesses different phenotypic characteristics, including virulence, at the strain level. To confirm our hypothesis, we isolated a *Malassezia* strain directly from the human intestinal mucosal surface of patients with UC and assessed its genome and virulence in comparison with those of the same fungal species isolated from the human skin. We successfully isolated live *M. globosa* strains from the intestinal mucosa of patients with UC. We compared the genomes, transcriptomes, and virulence of the *M. globosa* gut isolates with those of the *M. globosa* skin isolates to identify differences in genotypes and phenotypes caused by potential niche-specific adaptations of the fungus.

## RESULTS

### Mycobiota analysis identified that *Malassezia* resides in the mucosal layer of the human intestine in patients with UC

A study demonstrated that microbiomes in murine and human fecal samples only partially represent gut mucosal microbiomes (33). Moreover, previous mycobiome studies suggested that numerous fungi detected in fecal samples are temporal and originate from the environment, making it difficult to differentiate the true fungal colonizers in the gut (34, 35). Alternatively, analysis of mucosal layer-associated fungal communities provided more reliable data and revealed a strong correlation between the gut mycobiota and IBD, such as CD (25). Therefore, in this study, we first compared the mycobiota associated with the intestinal mucosal surface of patients with UC and healthy individuals by collecting water lavage samples during colonoscopy to investigate the presence and a potential role of *Malassezia* in the human gut environment, particularly in the context of IBD. Furthermore, to determine whether inflammation influences the composition of the mycobiota associated with the intestinal mucosal layer, we collected water lavage samples from the intestinal mucosal surfaces with and without inflammation from the same patient with UC. In addition, as mentioned earlier, we incubated a portion of each water lavage sample on a *Malassezia*-specific medium to isolate live *Malassezia* cells from the human gut mucosa.

**Fig 1:**
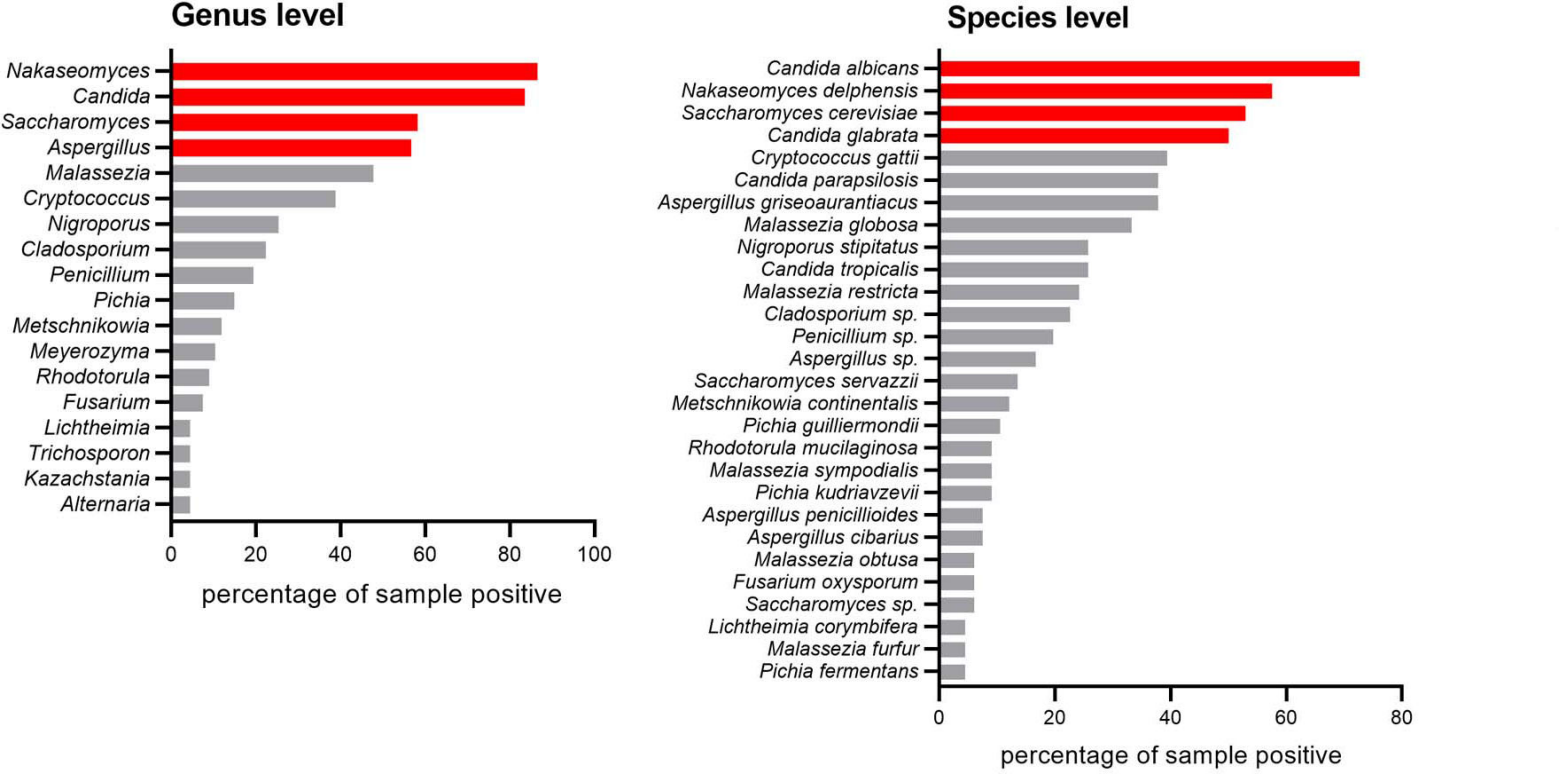
Results of amplicon sequencing analysis. Analysis of fungal genera and species most frequently present in all 67 lavage samples analyzed in this study. Fungal genera and species depicted in red bars are present in more than 50% of the lavage samples.

In total, 56 intestinal water lavage samples were obtained from 29 patients during colonoscopy. Of these, 54 were paired (one from the intestinal mucosal surface with inflammation and the other without inflammation from the same patient). Two samples were obtained only from the intestinal mucosal surface without inflammation from patients with UC. Moreover, 11 water lavage samples were obtained from 11 healthy individuals and included in this study. Detailed information on the samples is provided in Table 1 and Table S1. Once the water lavage samples (approximately 50 mL) were obtained, half of the samples were immediately inoculated on Leeming and Notman agar (LNA) medium within 3 h without freezing to minimize the loss of live fungal cells and incubated in the presence of 2% oxygen to mimic the intestinal environmental condition. The remaining samples were subjected to DNA extraction for amplicon sequencing. In total, 6,913,621 reads were obtained from 67 samples. After quality filtering and chimera removal, 6,621,891 reads were mapped to 1,074 amplicon sequence variants. *Nakaseomyces*, *Candida*, *Saccharomyces*, and *Aspergillus* were the top 4 abundant genera throughout the samples, and the fifth most abundant fungal genus in all samples was *Malassezia* (Fig. 1).

**TABLE 1:** Demographic and clinical characteristics of the participants

Characteristics	Patients with UC	Healthy controls
Number	29	11
Age (years)	48.5 ± 17.3	61.3 ± 9.9
Male sex, no. (%)	17 (58.6)	4 (36.4)
Disease extent (maximum), no. (%)		
Proctitis	2 (6.9)	–^*b*^
Left-sided colitis	8 (27.6)	–
Extensive colitis	19 (65.5)	
Endoscopic subscore, no. (%)		
0	3 (10.4)	–
1	7 (24.1)	–
2	16 (55.2)	–
3	3 (10.3)	
Sampling site, no. (%)		
With inflammation		
Rectum	4 (13.8)	–
Sigmoid colon	15 (51.7)	–
Descending colon	5 (17.2)	–
Transverse colon	0 (0.0)	–
Ascending colon	2 (7.0)	–
None	3 (10.3)	–
Without inflammation		
Rectum	4 (13.8)	0 (0.0)
Sigmoid colon	5 (17.2)	4 (36.3)
Descending colon	10 (34.5)	5 (45.5)
Transverse colon	8 (27.6)	0 (0.0)
Ascending colon	2 (6.9)	2 (18.2)
Concomitant medication (overlapped), no. (%)		
Immunomodulators	21 (72.4)	–
Prior use of biological agents	16 (55.2)	–
One medication	10 (62.5)	–
Two medications and above	6 (37.5)	–
Prior use of JAK inhibitors^*a*^	2 (6.9)	–

^*a*^ JAK inhibitors, Janus kinase inhibitors.

^*b*^ "–” represents either missing data or non-applicability.

We performed alpha and beta diversity analyses using the samples. Unlike our expectation, no significant differences were noted between patients with UC and healthy individuals or between sites with inflammation and those without inflammation at the intestinal mucosal surface of patients with UC (Fig. S1). Moreover, the relative abundance of all genera and species did not differ significantly between the groups (Fig. S2). A rank test was performed to determine whether the abundance of *Malassezia* is associated with UC. The abundance of *M. globosa* and *M. restricta* tended to be higher in patients with UC than in healthy individuals; however, the statistical significance of the difference was low (Fig. S3A). Moreover, no significant difference was noted in the abundance of both *M. globosa* and *M. restricta* between the intestinal mucosal surfaces with and without inflammation in each patient with UC, indicating a lack of correlation between the presence of inflammation and the abundance of *Malassezia* (Fig. S3B). Overall, although our data suggested no significant difference in the diversity of the mycobiota between patients with UC and healthy individuals and between mucosal samples with and without inflammation from the same patient, patients with UC tended to have a higher abundance of *M. globosa* and/or *M. restricta* in their gut mucosal surface than healthy individuals.

### Isolation of *M. globosa* from the intestinal mucosa and comparison with *M. globosa* skin isolates

In addition to performing mycobiota analysis by amplicon sequencing, water lavage samples were used to isolate live *Malassezia* strains from the intestinal mucosa. A total of 28 and 7 different species of live fungal strains were isolated from UC and healthy individuals, respectively (Table S2). Among these, two *M. globosa* strains were identified: one was isolated from the sigmoid colon without inflammation from patient no. 21 with UC, and the other was isolated from the same site but with inflammation from patient no. 50 with UC (Table S1). These two *M. globosa* strains were deposited at the Korean Collection for Type Cultures (KCTC; https://kctc.kribb.re.kr/en) and designated KCTC 37188 and KCTC 37189, respectively. Notably, both patients with UC displayed *M. globosa* reads in the amplicon sequencing data for mycobiota analysis.

**Fig 2:**
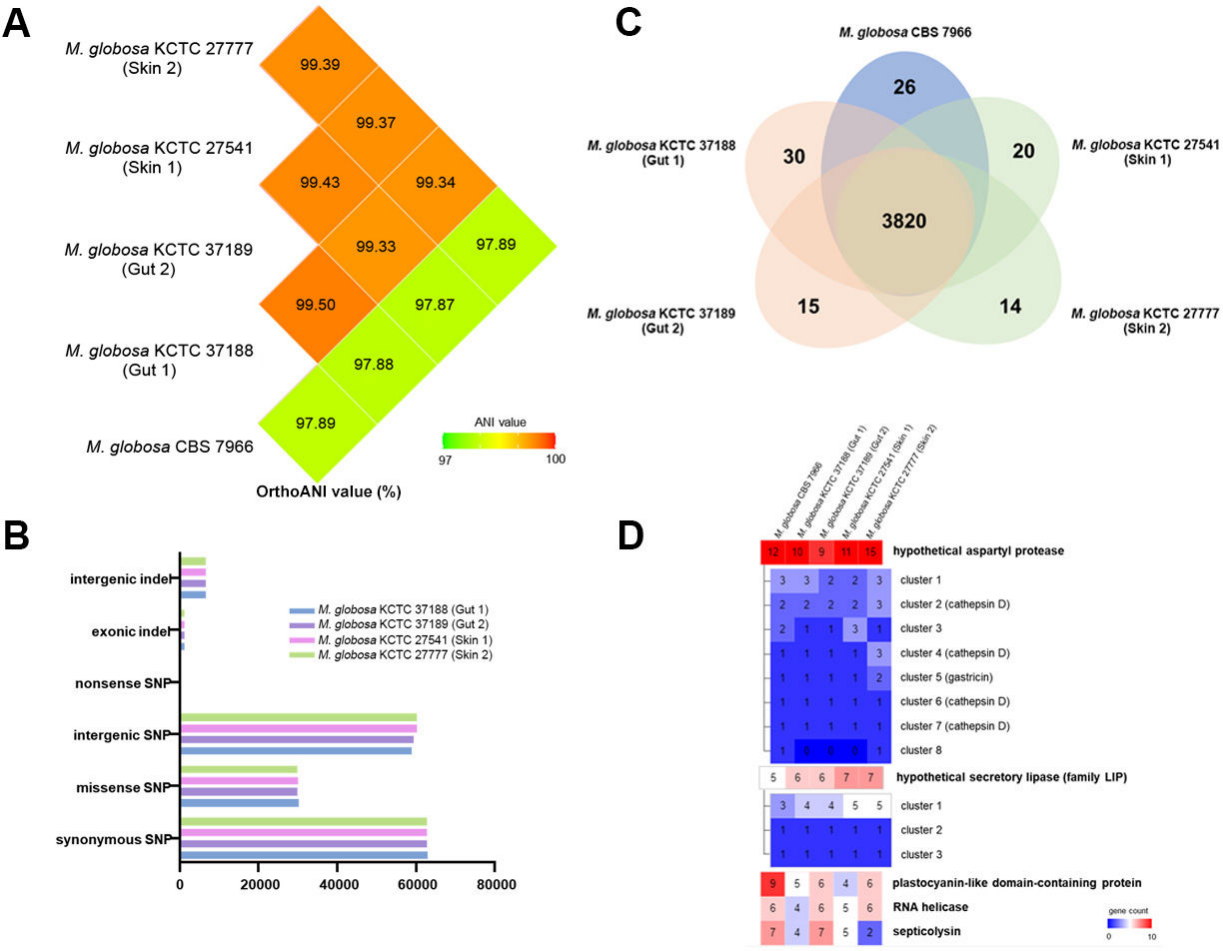
Genome comparison of *M. globosa* strains isolated from the gut and skin. (**A**) Comparison of ANI values between *M. globosa* strains. (**B**) A bar graph representing the results of SNP and indel analyses. The *x*-axis represents the number of indels and SNPs. (**C**) A Venn diagram showing the results of homologous clustering analysis, indicating the number of shared and unique genes in each strain. (**D**) A list of cluster groups, where the number of genes belonging to each orthologous cluster group differs among strains. The top 5 groups with the highest average number of genes were selected.

To determine whether *M. globosa* strains isolated from two different host niches, namely the gut mucosal surface and the skin, possess distinct characteristics, the genomes of the gut isolates *M. globosa* KCTC 37188 and KCTC 37189 and the skin isolates *M. globosa* KCTC 27541 and KCTC 27776, which were obtained from the forehead of patients with seborrheic dermatitis in our previous study (36), were sequenced using Illumina sequencing technology. Average nucleotide identity (ANI) was calculated based on the genome sequences of each strain and *M. globosa* CBS 7966, a type strain of the species that was isolated from the skin of a patient with pityriasis versicolor in England (https://wi.knaw.nl) (37). Overall, the ANI values between the gut and skin isolates were relatively high, indicating that the genome sequences of each strain were highly similar and exhibited no clear difference (Fig. 2A). Moreover, relatively low ANI values were observed between the *M. globosa* CBS 7966 type strain and the gut and skin isolates, possibly because of geological and ethnic differences in the isolation sites of the strains. A high similarity between the genomes of the gut and skin isolates was also confirmed by SNP and indel analyses on comparing the genomes with the reference genome of the type strain (Fig. 2B). Furthermore, homology-based gene clustering revealed that the gut and skin isolates and the type strain shared 3,820 common homologs (Fig. 2C). Few unique genes were identified in each strain: 30, 15, 20, 14, and 26 in the genomes of strains KCTC 37188, KCTC 37189 (gut isolates), KCTC 27541, KCTC 27777 (skin isolates), and CBS 7966, respectively. However, most unique genes in each strain were hypothetical and were predicted to produce a protein with an unknown function.

Previous studies on the genomes of multiple *Malassezia* species have suggested that *Malassezia* contains many genes that have high copy numbers. Most of these genes were predicted to contribute to the virulence of *Malassezia* and may be crucial for the survival of the fungus in the host environment. Examples include genes encoding lipases and proteases (38). In this study, we also analyzed genes with multiple copy numbers in the genomes of the gut and skin isolates and the type strain to identify the enrichment of any gene family in a specific isolate. The copy numbers of genes encoding protease and lipase were highly enriched across the strains, and no particular feature was observed (Fig. 2D). Moreover, we paid particular attention to the copy number of the gene encoding the homolog of septicolysin, which may be horizontally transferred from a bacterium (39), because of its putative endotoxin activity. The copy number of the septicolysin homolog gene varied across the strains; it was dependent on individual strains and not on the isolation site of each strain. Overall, no specific genomic characteristics were noted in the *M. globosa* gut isolates, especially in terms of nucleotide sequences and gene content levels, compared with the skin isolates and the type strain in this study.

### *M. globosa* isolated from the intestinal mucosal surface is more sensitive to oxygen than that isolated from the skin

**Fig 3:**
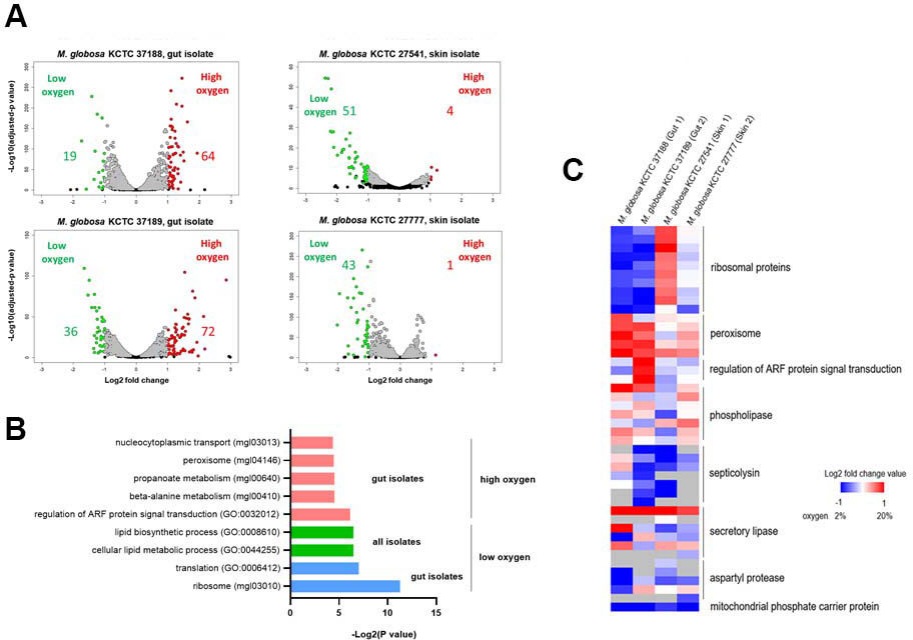
Comparison of transcriptomes of *M. globosa* strains isolated from the gut and skin. (**A**) Strains were grown under low-oxygen (2%) or high-oxygen (20%) conditions, and transcriptome profiles were analyzed by RNA-Seq. Volcano plots show the differential gene expression in fungal cells grown under high-oxygen conditions in comparison with those grown under low-oxygen conditions. Transcriptome analysis was performed in triplicate (three biological replicates), and only differentially expressed genes showing statistical significance (*P* < 0.05) were included in the analysis. Red: upregulated genes under low-oxygen conditions. Green: the number of downregulated genes under low-oxygen conditions. (**B**) Bar plots show the enrichment of GO terms and KEGG pathways in differentially expressed genes (DEGs) in each strain. GO enrichment analysis for the upregulated DEGs under high-oxygen conditions was conducted using only the genes obtained from the gut isolates, as the number of genes from the skin isolates was too small. Red: upregulated genes from gut-derived strains under high-oxygen conditions. Green: upregulated genes from both gut- and skin-derived strains under low-oxygen conditions. Blue: upregulated genes from gut-derived strains under low-oxygen conditions. (**C**) A heatmap of selected gene clusters from transcriptome analysis.

In addition to the genome contents, we compared the transcriptome profiles between the gut and skin isolates. We used different oxygen concentrations because oxygen plays a crucial role in the intestinal environment (40, 41) and hypothesized that the transcriptome profiles of the gut and skin isolates respond distinctly to different concentrations of oxygen. To compare the transcriptomes, we grew *M. globosa* gut and skin isolates in the presence of 2% and 20% oxygen, which likely represent the oxygen concentrations at the gut mucosal surface and skin surface, respectively, and analyzed and compared the transcriptome profile of each strain. Two independent gut isolates, KCTC 37188 and KCTC 37189, showed a relatively higher number of differentially expressed genes (83 and 108, respectively) than the skin isolates KCTC 27541 and KCTC 27777 (55 and 44, respectively) in response to increased oxygen concentrations (Fig. 3A). Particularly, more genes (64 and 72, respectively) were upregulated in the gut isolates, KCTC 37188 and KCTC 37189, in the presence of 20% oxygen, while only four and one genes were upregulated in the skin isolates KCTC 27541 and KCTC 27777 at the same condition, suggesting that the *M. restricta* gut isolates were more sensitive to higher oxygen conditions. We further analyzed the differentially expressed genes and found that five and three genes were commonly differentially expressed between the gut isolates, KCTC 37188 and KCTC 37189, in the presence of 2 and 20% oxygen conditions, respectively. However, these genes were annotated as hypothetical proteins; therefore, no meaningful information was obtained (Fig. S4).

We next conducted an enrichment analysis of KEGG pathways and GO terms of differentially regulated genes in each strain under different oxygen concentrations. In the gut isolates grown in the presence of 20% oxygen, highly enriched KEGG pathways and GO terms representing genes involved in peroxisome and ARF protein signal transduction were detected (Fig. 3B). As one of the major functions of peroxisomes is scavenging cells from oxidative stress, these results indicated the *M. globosa* gut isolates were under oxidative stress in the presence of 20% oxygen (42, 43). Furthermore, the homologs in the ARF protein signal transduction are involved in the regulation of stress responses via unfolded protein responses in *Saccharomyces cerevisiae*, supporting our finding that the *M. globosa* gut isolates were under stress in the presence of high concentrations of oxygen (44). In contrast, we did not note the enrichment of any specific gene family in the *M. globosa* skin isolates grown in the presence of 20% oxygen. When the *M. globosa* gut isolates were grown in the presence of 2% oxygen, genes involved in translation and ribosome functions were highly enriched, suggesting that the gut isolates were more actively growing at lower oxygen concentrations than the skin isolates. These results suggest that the *M. globosa* gut isolates are more sensitive to a higher oxygen concentration and exhibit better fitness at a lower oxygen concentration than the skin isolates.

Individual gene families that were specifically differentially regulated in the *M. globosa* gut isolates under the 20% oxygen condition compared with the 2% oxygen condition also included genes associated with ribosomal proteins and peroxisome pathways (Fig. 3C). In particular, the genes encoding ribosomal proteins were significantly downregulated in the gut isolates under the 20% oxygen condition, further confirming a decrease in protein synthesis and a resulting decrease in growth rate at a higher oxygen concentration. Moreover, the genes associated with the peroxisome pathway were highly upregulated in the gut isolates, also suggesting that the fungal cells are under oxidative stress and confirming the results of our KEGG and GO term enrichment analyses. To confirm our hypothesis based on the results from transcriptome analysis, we compared the growth of the gut and the skin isolates under two different oxygen concentrations. Two additional independent skin isolates, *M. globosa* KCTC 27523 and KCTC 27816, which were obtained in our previous study (36), were also included in addition to the skin isolates KCTC 27541 and KCTC 27777, which were used for transcriptome analysis, under two different oxygen concentrations to verify whether the gut isolate-specific phenotypes are indeed limited to gut isolates. The results showed the growth phenotypes of all four skin isolates were similarly distinct from the gut isolates under different oxygen concentrations: the slightly increased growth of the gut isolates was observed under the 2% oxygen condition relative to that in the 20% oxygen, confirming our observation (Fig. S5). Overall, transcriptome analysis suggested that the *M. globosa* gut isolates respond to oxygen concentrations differently from the skin isolates. Moreover, the results of transcriptome analysis suggested that the *M. globosa* gut isolates have evolved to adapt to the low-oxygen condition in the gut environment but are more sensitive to higher oxygen concentrations than the skin isolates.

### *M. globosa* isolated from the gut mucosa exacerbates DSS-induced colitis in mice

**Fig 4:**
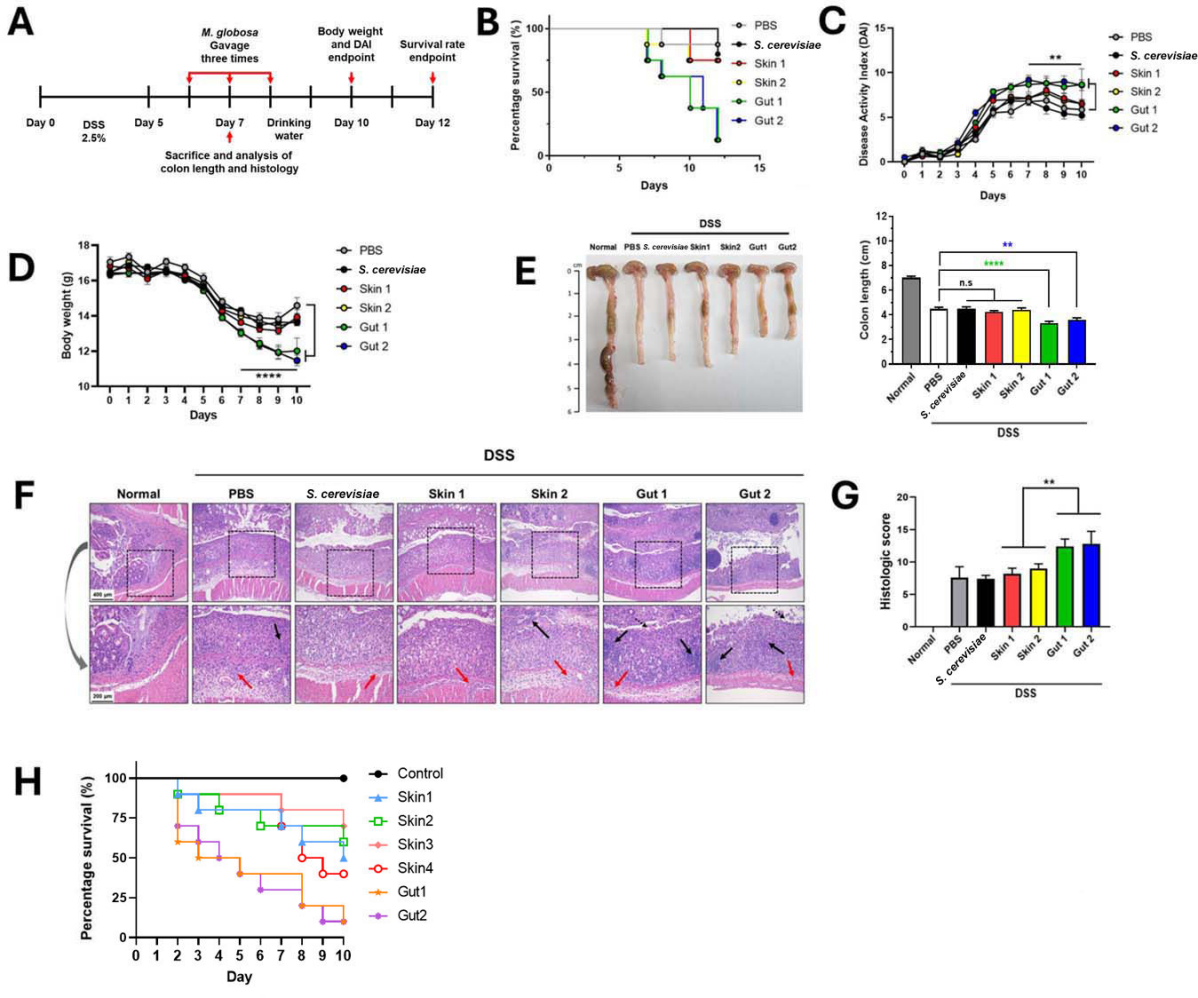
*M. globosa* isolated from the gut mucosa exacerbated DSS-induced colitis in mice. (**A**) Colitis was induced by DSS treatment for 5 days. *M. globosa* strains were orally gavaged a total of three times every other day, and the survival of mice and disease progression were monitored. (**B**) The survival of mice gavaged with each *M. globosa* strain was monitored. For each strain, 11 mice were infected. However, the reference strain *S. cerevisiae* ATCC 204508 was used to infect five mice. (**C**) Disease activity index (DAI) scores of mice gavaged with the indicated fungal strains. Each data point represents the mean ± standard deviations (SDs) of surviving animals at each time point. (**D**) Body weight loss of mice gavaged with the indicated fungal strains. Each data point represents the mean ± SDs of surviving animals at each time point. (**E**) Representative colons from mice with DSS-induced colitis gavaged with the indicated fungal strains. Colon lengths were measured on day 7 after sacrifice in five mice per group that were selected as representative based on DAI scores and histology results. (**F**) Representative hematoxylin and eosin-stained colon sections of mice with DSS-induced colitis gavaged with the indicated fungal strains. Black arrows indicate submucosal immune cell infiltrate, and red arrows indicate epithelial cell loss. (**G**) A bar chart representing averages (the mean values) with SDs of histological scores of at least five colon sections of mice sacrificed on day 7 gavaged with each indicated fungal strain control. (**H**) Results of survival assay using *Galleria mellonella*. Ten larvae were used for each strain to monitor survival rates. A 20 µL sample of each *M. globosa* strain (1.75 × 10^9^ cells/mL) was injected into the left proleg of the larvae, followed by incubation at 37°C in the dark. The control group was injected with 20 µL of 0.5% Tween 80. The survival of the larvae was monitored daily. **, *P* < 0.001.

We next investigated whether the *M. globosa* gut isolates are associated with exacerbating colitis in the mouse model and compared their pathogenesis with those of the skin isolates. We treated the mice with DSS for 5 days to induce colitis. After DSS treatment, we inoculated the fungal cells of each strain by oral gavage every other day for a total of three times and monitored the disease progress (Fig. 4A). We also compared the virulence and contribution of the gut isolates to colitis with those of the skin isolates to determine whether strains from different host niches influence the disease.

Mice inoculated with two independent gut isolates showed a significantly reduced survival rate; 9 out of 11 mice were dead on day 7 postinfection. In contrast, most mice inoculated with two independent skin isolates survived; only 2 out of 11 mice were dead (Fig. 4B). The negative control and the reference strain *S. cerevisiae* ATCC 204508 showed similar results to the skin isolates, confirming that only the gut isolates and not the skin isolates influenced the survival of the mice.

In addition to the survival assay, we conducted separate experiments to determine the disease activity index (DAI) of colitis, as described previously (45). Notably, we reduced the number of oral gavages of fungal cells to mice with DSS-induced colitis from three to two in order to maintain the survival of the host during the experiments. We found that the DAI score of the mice gavaged with the negative control, the *S. cerevisiae* reference strain, and the *M. globosa* skin isolates quickly reduced in 2–3 days after oral gavage of the fungal cells. In contrast, the DAI scores of the mice inoculated with the *M. globosa* gut isolates were relatively higher than those of the other groups (Fig. 4C). The DAI scores revealed that body weight loss was significantly greater, and colon length was significantly lower in the mice gavaged with the *M. globosa* gut isolates than in the other groups (Fig. 4D and E). Moreover, histological scoring revealed that the symptoms of colitis were greater in the mice gavaged with the *M. globosa* gut isolates than in the other groups (Fig. 4F and G). We also carried out an *in vivo* competition assay to compare the survival of the gut and the skin isolates. Equal amounts of all four isolates, two gut and two skin isolates, were mixed and orally gavaged into a DSS-induced colitis mouse, and the presence of each strain in the gut was monitored daily by detecting DNA of the gut or the skin isolates in feces using a real-time quantitative polymerase chain reaction (qPCR) (46). As shown in Fig. S6, the results indicated that a significantly higher amount of the gut isolates was detected in feces until day 4 compared to the skin isolates, which were observed up to day 2. These results suggested that the *M. globosa* gut isolates survived longer in the gut than the skin isolates.

Different pathogenicities of the *M. globosa* gut isolates compared to the skin isolates were also assessed in *Galleria mellonella* (greater wax moth), a model system that resembles the mammalian gut environmental conditions (47, 48), to confirm the results using the mouse colitis model. *G. mellonella* larvae and mammalian gastrointestinal tracts share similar tissues, anatomy, and physiological functions and conditions. *G. mellonella* can also survive at a temperature of 37°C. We injected each fungal strain into the larvae, incubated the larvae at 37°C, and monitored larval survival daily. Two additional skin isolates, *M. globosa* KCTC 27523 and KCTC 27816, which were obtained in our previous study, were also included in this survival assay. The larvae injected with the *M. globosa* gut isolates showed a significantly reduced survival rate (Fig. 4H). Moreover, survival of each fungal strain within the larvae was monitored daily by detecting DNA of the gut or the skin isolates in the homogenized larvae using qPCR. The significantly higher amount of the gut isolates was detected in the larvae up to 10 days, while the amount of the skin isolates started to reduce after day 7, suggesting that the *M. globosa* gut isolates survived longer in the *G. mellonella* larvae than the skin isolates (Fig. S7). These results further support our finding that the *M. globosa* gut isolates are more suitable under mammalian gut environment-like conditions than the skin isolates.

**Fig 5:**
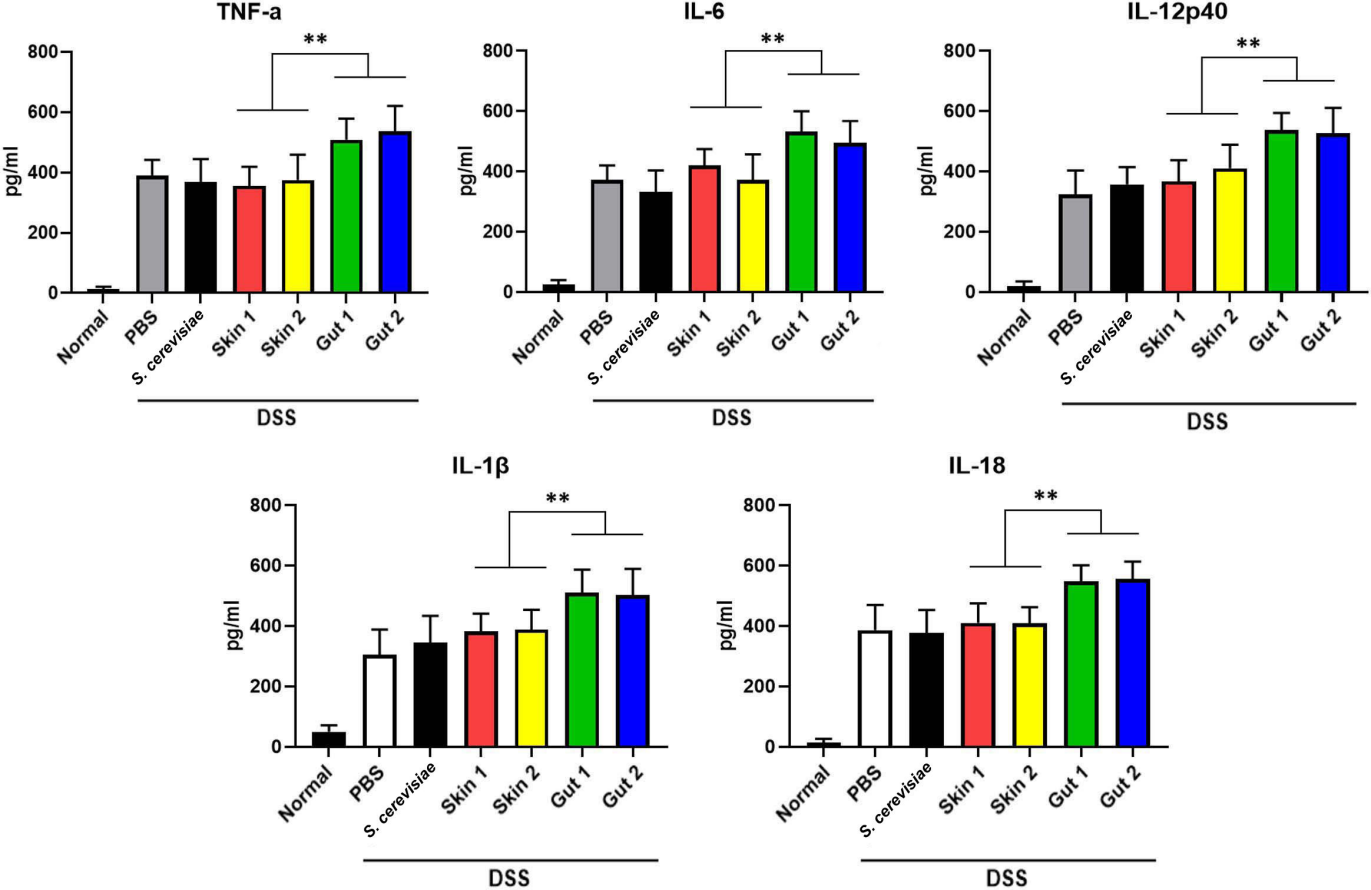
Levels of cytokines in *M. globosa*-associated colitis in mice. Cytokine levels were measured in colonic tissue extracts obtained from five mice sacrificed on day 7. These mice were selected as representative based on their DAI scores and colon histology results. Values represent the averages (the mean values) of cytokine levels measured from five independent colonic tissue extracts for each fungal strain. Error bars indicate SDs (**P < 0.005). Reference, *S. cerevisiae* ATCC 204508; Skin 1, *M. globosa* KCTC 27541; Skin 2, *M. globosa* KCTC 27777; Gut 1, *M. globosa* KCTC 37188; and Gut 2, *M. globosa* KCTC 37189.

Maintaining a proper balance of inflammatory cytokines is crucial for a healthy gut homeostasis, and the disruption of this balance can trigger the progression of diseases, including UC. During the activation of inflammatory cascades in the gut mucosa of patients with UC, the levels of various inflammatory cytokines are inappropriately elevated (49, 50). Therefore, we measured the levels of proinflammatory cytokines, including TNF-α, IL-6, IL-12p40, IL-1β, and IL-18, in the mice gavaged with the *M. globosa* gut isolates. The results of the enzyme-linked immunosorbent assay (ELISA) indicated that the cytokine levels were significantly higher in the mice inoculated with the *M. globosa* gut isolates than in those inoculated with the *M. globosa* skin isolates and the *S. cerevisiae* reference strain (Fig. 5). These results further confirmed that the *M. globosa* gut isolates and not the skin isolates could affect inflammation and exacerbate DSS-induced colitis in the mouse model.

## DISCUSSION

In general, unlike bacterial diversities, fungal diversities in the gut of healthy individuals and patients with IBD exhibit significant individual variations. Moreover, different conclusions have been reported in different studies, which may be influenced by the methodologies used and the regions and diets of the analyzed cohorts (51, 52). Nevertheless, a few common features of the gut mycobiota in patients with IBD, such as an increased overall fungal load and an increased Basidiomycota/Ascomycota ratio, have been reported (53, 54). Furthermore, several studies have reported a distinct *Malassezia* population in the gut of patients with IBD, indicating a possible role of the fungus in IBD (21, 25, 53–56). However, in our study, no statistically significant difference was noted in fungal diversity between the gut mucosa of patients with UC and healthy individuals. Similarly, a recent study using fecal samples from a Japanese cohort revealed no significant differences in fungal diversity between patients with UC and healthy controls (56), implying that the structure of the gut mycobiota in East Asian patients with UC, in particular, is similar to that of healthy individuals.

Despite the overall similarity of mycobiome structures between UC and healthy individuals, we aimed to isolate and investigate the roles of *Malassezia* in the human gut environment, because previous studies have suggested the influence of the fungus on gut health as mentioned above (21, 25, 53–56). Two independent live *M. globosa* strains were isolated directly from the gut mucosa of two independent UC patients. It should be noted that we failed to obtain *M. restricta*, although fungal community analysis using amplicon sequence reads indicated a significant amount of *M. restricta* in the gut mucosa of UC patients. Failure to isolate *M. restricta* directly from the gut mucosa implies a distinct requirement of a specific nutrient and/or culture condition for the *M. restricta* species associated with the gut mucosa in comparison with the same species isolated from the skin. This also suggests a possible intraspecies variant of the fungus. Nevertheless, our data suggest that the *M. globosa* strains directly isolated from the gut mucosa of patients with UC exacerbate DSS-induced colitis in the mouse model. The increased disease severity was supported by the findings of increased DAI and histopathological scores, decreased gut length, and increased production of inflammatory cytokines in mice inoculated with *M. globosa* gut isolates.

The existence of intraspecies variants has been reported in major human pathogenic fungi, such as *Cryptococcus neoformans* and *Candida albicans*. Analysis of 230 clinical isolates of *C. neoformans* from patients with HIV in South Africa revealed a close association of genotypes and phenotypes, including virulence traits of the strains, with clinical outcomes (57). Clinical isolates of *C. albicans* also exhibited large genetic diversity, resulting in phenotypic differences between the strains, including host immune responsiveness and pathogenicity (58–60). In addition, niche-specific adaptation of *C. albicans* strain has been suggested. Lemberg et al. compared the transcriptomes, metabolic profiles, and pathogenicity between two *C. albicans* strains, namely strain 101, which was isolated from the oral mucosa of a healthy individual, and SC5314, a well-known laboratory standard reference strain that is also known as a hypervirulent strain. *C. albicans* strain 101 exhibited low virulence and had metabolically adapted to the oral epithelium to persist as a commensal, while the highly virulent strain SC5314 induced strong inflammation (61). Considering these findings, we compared the genome sequences of *M. globosa* strains that were directly isolated from the gut mucosa of patients with UC in this study with those of *M. globosa* strains isolated from the skin in our previous study (62). Our data revealed no obvious differences in genome sequences between the gut and skin isolates. Comparisons of gene copy number variations (CNVs) showed enrichment of genes involved in stress response and virulence in all *M. globosa* isolates, but no difference was noted between the strains.

CNV influences the tolerance of cells to stressful environments. In genetic studies using *S. cerevisiae*, CNV has been found to affect gene expression and cause the overexpression of a corresponding gene, directly influencing the phenotypes of cells. If the benefits outweigh the cost, CNVs become fixed; the underlying mechanism is highly specific to each strain (63). Although our data suggested no difference in CNV between the gut and skin isolates, genome analysis revealed that many genes showed significant CNV across the strains. Genes showing significant CNV are likely essential players involved in stress tolerance, survival, and virulence of *M. globosa*, especially in the host environment. The top 3 genes that showed significant CNV across the strains included those associated with iron transport, RNA helicase, and the bacterial enterotoxin septicolysin homolog. Iron transport plays a vital role in stress tolerance and virulence of pathogenic fungi (64). RNA helicase is involved in stress tolerance, and its homologous protein, Ski2, in *C. neoformans* plays a key role in conferring resistance to azole antifungal drugs and tolerance to stressful conditions (65). CNV of the gene encoding the homolog of septicolysin, which may act as a bacterial cytolytic toxin in mammalian hosts (66, 67), was observed across the strains, suggesting that the gene may play a role in *M. globosa* in the host niche.

Maintaining a proper gut environment is essential for the prevention of disease. The gut microbiota, including the mycobiota, plays a critical role in gut homeostasis. Oxygen is a microenvironmental factor that contributes to homeostasis in the gut ecosystem and influences the metabolism and immunity of the gut and the colonization of pathogens. Several studies have demonstrated that disruption of the oxygen gradient significantly contributes to various intestinal diseases such as IBD and colorectal cancer (40, 41). The existing oxygen gradient has been reported to be 5%–10% at the crypt and lumen interface and approximately 2% and 0.4% in the lumen of the ascending and sigmoid colon, respectively (40, 68). Multiple mechanisms are involved in the reduction of oxygen concentrations in the gut. These include microbially derived short-chain fatty acids such as butyrate, which can promote mitochondrial beta oxidation and oxidative phosphorylation in colonocytes to increase oxygen consumption and induce a hypoxic environment in the gut, thereby suppressing the colonization of pathogenic bacteria such as *E. coli* and *Salmonella*. In contrast, antibiotic treatment increases oxygen concentrations, reprograms colonocytes to a state of anaerobic glycolysis from oxidative metabolism, and triggers an increase in the population of facultative pathogenic bacteria such as *Salmonella* (69, 70).

Compared with hypoxic conditions in the gut, normoxia is observed in the skin, especially the outermost surface of the skin where *Malassezia* predominantly resides. Therefore, we hypothesized that the *M. globosa* gut and skin isolates have distinctly adapted to environments with different oxygen concentrations and display different physiological responses to low and high concentrations of oxygen. For example, the *M. globosa* gut isolates may be more sensitive to normoxia than the skin isolates are. To confirm this hypothesis, we analyzed and compared the transcriptomes of the *M. globosa* gut and skin isolates that were grown in the presence of 2% or 20% oxygen. A comparison of growth between the *M. globosa* gut and skin isolates under different oxygen concentrations was performed, and the results confirmed that the *M. globosa* gut isolates grew slowly in the presence of 20% oxygen compared to 2% oxygen. The longer survival of the *M. globosa* gut isolates within *Galleria* larvae further supports our hypothesis. Transcriptome analysis revealed that the genes involved in peroxisome functions and stress responses were upregulated in the *M. globosa* gut isolates in the presence of 20% oxygen, indicating that the fungal cells were highly responsive to oxygen concentrations and experienced oxidative stress under 20% oxygen conditions compared to the skin isolates. The enrichment of genes involved in ribosomal functions in the *M. globosa* gut isolates in the presence of 2% oxygen further supported our finding that the gut isolates may prefer a low-oxygen environment, which may resemble the environment at the gut mucosal surface.

The different transcriptome profiles observed between the gut and skin isolates may reflect the phenotypes of the strains. The *M. globosa* gut isolates showed distinct virulence levels compared with the skin isolates. The gut isolates exacerbated DSS-induced colitis in the mouse model, while the skin isolates showed outcomes similar to those of the negative control and the reference strain *S. cerevisiae*. Thus, although they are the same species, the *M. globosa* gut and skin isolates may have adapted to different environmental niches in the host body and may have thereby gained unique pathological characteristics.

Intraspecies diversity within the same fungal species has been commonly reported in recent studies (58, 71–73). For example, *S. cerevisiae* and *C. albicans* isolated from the feces of patients with CD displayed different *in vitro* and *in vivo* immune responses (72). Moreover, *C. albicans* strains isolated from patients with irritable bowel syndrome (IBS) displayed phenotypic diversities, and genotypic analysis revealed that the strains isolated from the gut of hypersensitive IBS patients partially clustered together (73). However, whether the colonization of a specific body site is associated with genotypic and phenotypic diversities, including virulence, remains unclear. A recent study using 910 commensal isolates from 35 healthy donors reported extensive phenotypic diversity that was independent of the isolation site or participant (60). Furthermore, high rates of heterozygosity, structural mutations, and chromosomal variations in *C. albicans* have been reported in other studies (74–76). Unlike *C. albicans*, we found that the genotypes and phenotypes of *M. globosa* clinical isolates were associated with the body site, although a limited number of the fungal isolates were used. Moreover, to date, no information has been provided on the degree of heterozygosity, structural mutations, and chromosomal variation using a reasonable number of *M. globosa* clinical isolates. Therefore, further research should be conducted to analyze and confirm the relationship between a specific body site and genotypic and phenotypic variations in *Malassezia* species using a large number of clinical isolates.

In conclusion, our findings demonstrate that *M. globosa* strains isolated from the gut mucosa of UC patients exhibit distinct characteristics from skin isolates, including increased virulence in the colitis mouse model and differential responses to oxygen levels. Our results highlight the complexity of host-fungal interactions in IBD and suggest that niche-specific fungal adaptations should be considered to better understand these interactions.

## MATERIALS AND METHODS

### Fungal strains

*S. cerevisiae* ATCC 204508 was used as a negative reference strain and grown overnight at 30°C in yeast extract peptone dextrose (YPD) medium. *M. globosa* KCTC 27541, KCTC 27777, KCTC 27523, and KCTC 27816, which were isolated from the skin lesions on the scalp of patients with seborrheic dermatitis in our previous study (36), were used as skin isolates and compared with the gut isolates throughout the study. Two independent *M. globosa* strains were isolated from the gut mucosa of two different patients with UC during colonoscopy, as described below. The strains were identified by polymerase chain reaction (PCR) using the primers ITS4 (TCCTCCGCTTATTGATATGC) and ITS5 (GGAAGTAAAAGTCGTAACAAGG) and by sequencing. They were deposited at the KCTC and designated *M. globosa* KCTC 37188 and KCTC 37189, respectively (77). *M. globosa* strains were cultured at 34°C on LNA medium for 3 days and used throughout the study.

### Collection of mucosal lavage samples

This study was conducted in adult patients with UC who were aged 19 years and older and attended Chung-Ang University Hospital between October 2021 and August 2022 and healthy controls. Samples from patients with UC were obtained during colonoscopic examinations for clinical reasons. In total, 20 mL of distilled water was injected twice or thrice into the colonic mucosa through the instrument channel of a colonoscope, followed by collection (approximately 50 mL) through the suction channel. These mucosal lavage samples were collected separately from areas with and without inflammation from the same patient. The definition of a non-inflamed gut area was based on the Mayo endoscopic score (78), referring to regions where no endoscopic signs of inflammation were observed. Specifically, it was characterized by a normal vascular pattern without erythema, friability, erosions, ulcers, or bleeding, indicating an endoscopically normal segment. To minimize potential cross-contamination, lavage was performed at least 30 cm away from any inflamed area. Care was taken to avoid mixing samples from different gut sections, ensuring that each lavage sample corresponded to a well-defined anatomical region. These precautions ensured the spatial distinction of the lavage samples, allowing for a reliable comparison between inflamed and non-inflamed areas. Patients’ clinical data, including demographic factors, disease characteristics, endoscopic findings at the time of sample collection, and sampling sites, were also recorded. The surgical history of bowel resection and medication history, including the use of steroids, immunomodulators, and biologics, was also recorded. Healthy controls were individuals who visited the gastroenterology clinic to undergo colonoscopy for routine check-ups and had not been diagnosed with CD, UC, or functional gastrointestinal disorders based on the ROME IV criteria (79). Participants with serious chronic conditions that could potentially impact the results of this study, such as uncontrolled hypertension, diabetes, pulmonary tuberculosis, liver cirrhosis, and chronic kidney disease, as well as those who had received systemic or local antifungal agents within the past month, were excluded. Mucosal lavage samples were obtained from healthy controls using the same procedure as that used for patients with UC; the samples were obtained from a randomly selected area of the colon during routine colonoscopic examinations.

### DNA extraction and fungal isolation

The mucosal lavage samples were centrifuged at 6,000 rpm for 30 min at room temperature, and the pellet was suspended in 1 mL of phosphate-buffered saline (PBS). DNA was extracted from 500 µL of the suspension, as described previously (80), and used for fungal community analysis. The remaining suspension was spread on LNA medium containing antibiotics (gentamicin 100 µg/mL, chloramphenicol 50 µg/mL) to restrict the growth of bacterial cells and cultured for 2 weeks at 34°C in the presence of 2% oxygen. A single colony was obtained and subjected to species identification by PCR amplification using ITS4 and ITS5 primers and by sequencing, as described above.

### Library construction, sequencing, and data analysis of the mycobiome

Sequencing libraries were constructed to amplify the ITS2 regions using Illumina’s Metagenomic Sequencing Library protocols using ITS3 and ITS4 primers, 5′-TCGTCGGCAGCGTCAGATGTGTATAAGAGACAGGCATCGATGAAGAACGCAGC-3′ and 5′-GTCTCGTGGGCTCGGAGATGTGTATAAGAGACAGTCCTCCGCTTATTGATATGC-3′, respectively, as described in our previous study (81). Raw sequence reads were subjected to quality control, including trimming and adapter removal using Trimmomatic v.0.36 (82). Paired-end reads were merged with PEAR v.0.9.6 (83), and subsequently imported into the QIIME 2 pipeline v.2020.8 (84). Targeted Host-associated Fungi v.1.6.1 mycobiome database was used for fungal taxonomic classification (85). Alpha and beta diversity metrics were calculated within QIIME 2 (84). Negative controls, consisting of blank PBS solutions, were sequenced to ensure the absence of contamination in the reagents.

### Genome sequencing and analysis

Genomic DNA of the *M. globosa* gut isolates (KCTC 37188 and KCTC 37189) and skin isolates (KCTC 27541 and KCTC 27777) was extracted using the Wizard HMW DNA Extraction Kit (Promega, Madison, WI, USA), according to the manufacturer’s recommendations. The sequencing library was constructed using the TruSeq DNA PCR-Free Library Prep Kit (Illumina, San Diego, CA, USA), according to the manufacturer’s instructions. NGS was performed using NovaSeq 6000 (Illumina, San Diego, CA, USA) on a 151 bp paired-end platform. The generated raw data were quality filtered and adapter trimmed by Trimmomatic v.0.36 (82) using default parameters. Cleaned reads were assembled using the CLC Genomics Workbench v.20 (Qiagen, Germantown, MD, USA). From the resulting contigs, gene prediction and annotation were performed by FunGap v.1.1.0 (86) using the RNA sequencing (RNA-Seq) data of each strain. Orthologs were grouped by DIAMOND v.2.0.6 (87) with the parameter “-id 50” and MCL 14-137 with the parameter “-I 1.5” (88). ANI values were analyzed using the ANIb method based on BLAST via JSpecies-WS (89).

### RNA extraction and transcriptome analysis

*M. globosa* strains were cultured in LNA medium for 3 days at 34°C in the presence of 2% or 20% oxygen (CO_2_/O_2_ incubator, Vision Scientific, Daejun, Korea). RNA extraction was performed using TRIzol Reagent (Invitrogen, Carlsbad, CA, USA), according to the manufacturer’s instructions. The extracted RNA was further treated using the TURBO DNA-Free Kit (Ambion, Austin, TX, USA), according to the manufacturer’s instructions. Sequencing libraries for RNA-Seq were generated following the manufacturer’s protocols for the Illumina TruSeq Stranded mRNA Library Prep Kit (Illumina, San Diego, CA, USA). Sequencing was performed using NovaSeq 6000 (Illumina, San Diego, CA, USA), according to the manufacturer’s instructions, and 101 bp paired-end reads were generated. Raw data were trimmed using the same procedure as that used for genome sequencing. Cleaned reads were mapped to the assembled genome using HISAT2 v.2.2.1 (90). The reads that were mapped to each CDS were counted using featureCounts (91). Finally, the counts from each CDS were normalized and statistically analyzed using the DeSeq2 package (92). Functional enrichment analysis against differentially expressed genes under each condition was performed using DAVID Bioinformatics Resources (93).

### *Galleria mellonella* survival assay

Upon arrival, the larvae were fed with sawdust and stored at room temperature for 1 h to facilitate recovery before infection. Larvae that weighed between 250 and 330 mg were sized between 2 and 2.5 cm, and those that did not have dark dots were selected and cleaned with 70% ethanol. Ten larvae were used for each strain to monitor survival rates. A 20 µL sample of each *M. globosa* strain (1.75 × 10^9^ cells/mL) was injected into the left proleg of the larvae, followed by incubation at 37°C in the dark. The control group was injected with 20 µL of 0.5% Tween 80. The survival of the larvae was monitored daily. The larvae were considered dead if they did not respond to tactile stimulation (94). The survival curve was generated using the GraphPad Prism program by Dotmatics, employing the Kaplan-Meier survival curve method. The log-rank test (Mantel-Cox) was employed to compare differences between groups. Statistical significance was determined at a *P*-value ≤0.05.

### Determination of survival rate and DAI using a DSS-induced colitis mouse model

*M. globosa* gut isolates (KCTC 37188 and KCTC 37189) and skin isolates (KCTC 27541 and KCTC 27777) were cultured on LNA medium for 3 days at 34°C. *S. cerevisiae* ATCC 204508 was cultured on YPD agar for 2 days at 30°C. The fungal cells were scraped from the agar plate, washed in PBS, and filtered using a 40 µm-diameter filter. The filtered cells were then counted and resuspended to a concentration of 1 × 10^8^ fungal cells/mL. Eleven female C57BL/6 mice (Koatech, Pyeongtaek, Korea) per group were used.

A DSS-induced colitis mouse model was developed using 6-week-old female C57BL/6 mice (Koatech, Pyeongtaek, Korea), as described previously (95). Colitis was induced using 2.5% DSS (molecular weight: 36,000–50,000 kDa, MP Biomedicals, Santa Ana, CA, USA) for 5 days, followed by water for 2 days (i.e., a total of 7 days). On day 8, 100 µL of PBS containing 1 × 10^7^ fungal cells was orally gavaged to the colitis-induced mice every other day for 5 days (three injections in total), and the survival of the mice was monitored daily. Five mice were sacrificed on day 4 after two injections, and the colon length and DAI score were determined as reported previously (45). During the experiment, the stool and weight of each mouse were assessed and scored daily.

### ELISA

The levels of TNF-α, IL-6, IL-12p40, IL-1β, and IL-18 were measured in mouse colonic tissue extracts by using the BD OptEIA ELISA System (BD Biosciences, San Jose, CA, USA). All assays were performed as per the manufacturer’s instructions.

### Histological assessment

For immunohistochemical assessment of tissue sections, the distal colonic tissues of mice were fixed in 10% formalin and embedded in paraffin. The paraffin-embedded sections (4 µm thick) were sliced and stained with hematoxylin and eosin. Histopathological scores and H-scores (a semiquantitative measure) were assessed (96). For this purpose, a board-certified pathologist (Dr. Min-Kyung Kim, Kim Min-Kyung Pathology Clinic, Seoul, Korea) independently evaluated each organ section without prior knowledge of the treatment groups.

### Competition assay

Colitis was induced in mice using DSS, as described above. In total, 100 µL of PBS samples containing a mixture of *M. globosa* gut isolates (KCTC 37188 and KCTC 37189) and skin isolates (KCTC 27541 and KCTC 27777) (1 × 10^7^ cells of each strain) were orally gavaged once into the colitis-induced mice. The feces of the mice were harvested on a daily basis. Fecal DNA was extracted using the AllPrep Bacterial/Fungal DNA/RNA/Protein Kit (Qiagen, Germantown, MD, USA), and *M. globosa* strains were detected using qPCR. In brief, qPCR was performed using gut isolate- or skin isolate-specific primer sets, input gDNA (50 ng), and SYBR Green PCR Master Mix (Roche, Mississauga, ON, USA) on a QuantStudio 3 system (Thermo Fisher Scientific, Waltham, MA, USA), according to the manufacturer’s instructions. The PCR program consisted of 40 cycles of 15 s at 95°C, 15 s at 56°C, and 45 s at 72°C. The sequences of the primers used were as follows: skin isolate-specific forward, 5′-CTTTCTCCTCAAGCTGGCG-3′; skin isolate-specific reverse, 5′-GGGCTTGGCACTTTGAACAT-3′; gut isolate-specific forward, 5′-CTTTCTCCTCAAGCTGGCA-3′; and gut isolate-specific reverse, 5′-GGGCTTGGCACTTTGAACAT-

### Statistical analysis

Statistical differences between groups were evaluated using one-way analysis of variance with Tukey’s multiple comparison test on GraphPad Prism version 9.5.1. Statistical significance was defined as a *P* value ≤0.05.

## Data Availability

Whole genome shotgun projects have been deposited in DDBJ/ENA/GenBank under the accession numbers JAZHEV000000000, JAZHEW000000000, JAZHEX000000000, and JAZHEY000000000. Transcriptome data were deposited in GEO under the accession number GSE254809. Microbiome amplicon sequencing data were deposited in SRA database under the accession number PRJNA1070443.

## SUPPLEMENTAL MATERIAL

Figure S1 mbio.01400-25-s0001.pdf Alpha- and beta-diversity analyses.

Figure S2 mbio.01400-25-s0002.pdf Bar plots of species-level abundance in mycobiome data sets.

Figure S3 mbio.01400-25-s0003.pdf The results of the rank test.

Figure S4 mbio.01400-25-s0004.pdf Venn diagrams to determine common genes showing differential regulation between isolates.

Figure S5 mbio.01400-25-s0005.pdf Growth comparison assay.

Figure S6 mbio.01400-25-s0006.pdf *In vivo* competition assay.

Figure S7 mbio.01400-25-s0007.pdf Relative quantification of *M. globosa* DNA in *G. mellonella* larvae.

Table S1 mbio.01400-25-s0008.pdf List of patients (healthy controls and patients with UC): age, sex, disease information, and treatment data.

Table S2 mbio.01400-25-s0009.pdf Live fungal strains obtained in this study.

ASM does not own the copyrights to Supplemental Material that may be linked to, or accessed through, an article. The authors have granted ASM a non-exclusive, world-wide license to publish the Supplemental Material files. Please contact the corresponding author directly for reuse.
