# Supplementary material for: Live Malassezia strains from the mucosa of patients with ulcerative colitis: pathogenic potential and environmental adaptations
Source: mBio. 2025 Jun 13;16(7):e01400-25. doi: 10.1128/mbio.01400-25 (PMC12239588; doi:10.1128/mbio.01400-25)
Supplement: Figure S1 — Alpha- and beta-diversity analyses. [file mbio.01400-25-s0001.pdf]

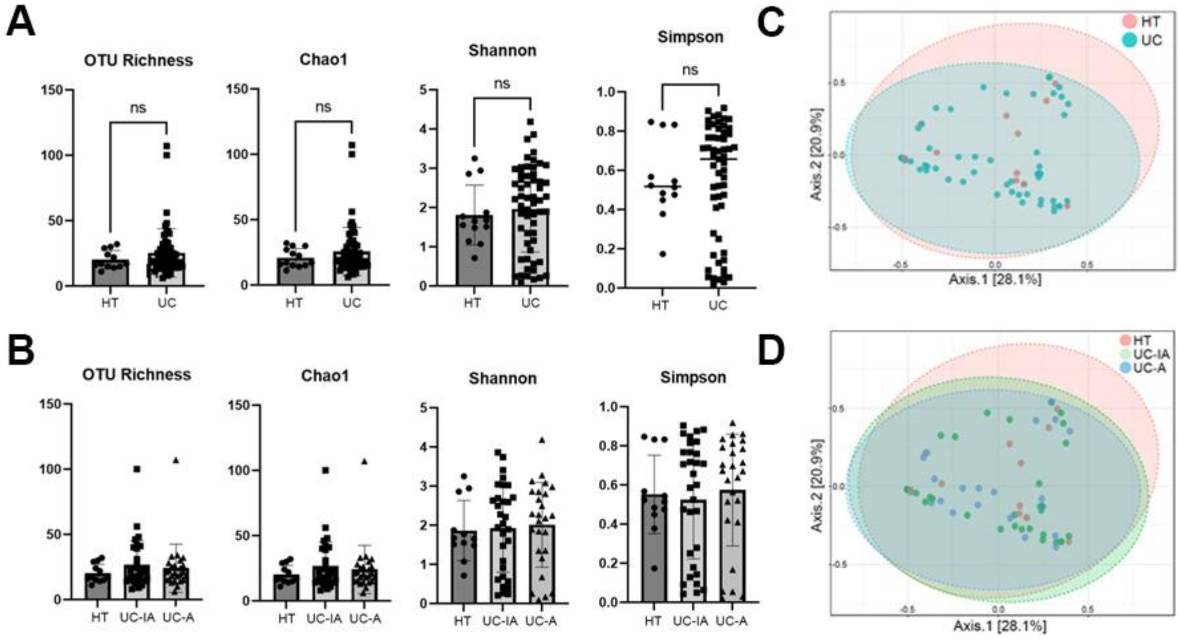

Fig. S1. α- and β-diversity analyses. Box plots of α-diversity distribution (richness and Chao, Shannon, and Simpson diversities). **A.** Healthy controls (HT) versus patients with UC. **B.** Healthy controls (HT) and the sites with inflammation (UC-A) versus those without inflammation (UC-IA) from patients with UC. PCoA plots of β-diversity. **C.** Healthy controls (HT) versus patients with UC. **D.** Healthy controls (HT) and the sites with inflammation (UC-A) versus those without inflammation (UC-IA) from patients with UC.
