# Supplementary material for: Live Malassezia strains from the mucosa of patients with ulcerative colitis: pathogenic potential and environmental adaptations
Source: mBio. 2025 Jun 13;16(7):e01400-25. doi: 10.1128/mbio.01400-25 (PMC12239588; doi:10.1128/mbio.01400-25)
Supplement: Figure S2 — Bar plots of species-level abundance in mycobiome data sets. [file mbio.01400-25-s0002.pdf]

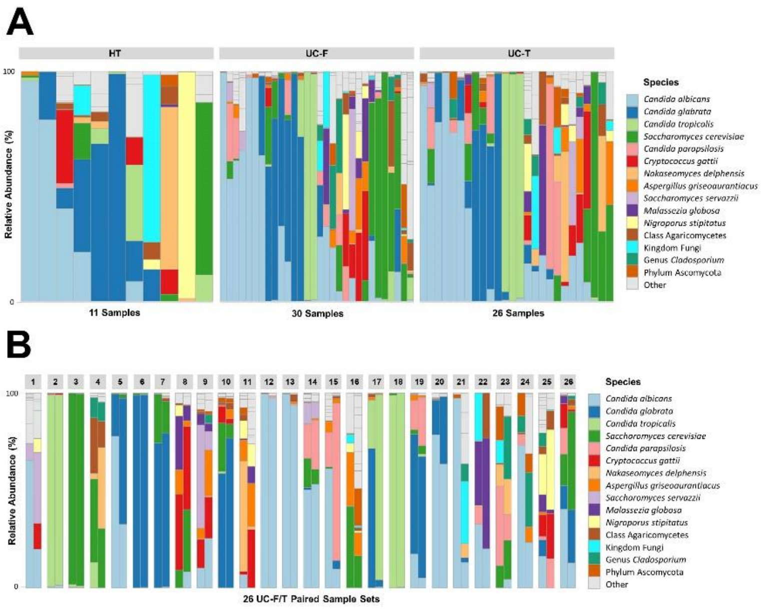


**Fig. S2**. Bar plots of species-level abundance in mycobiome datasets. **A.** Taxa bar plot of species-level mycobiome identified by ITS amplicon sequencing across all samples. The top 15 species are shown in the order of average abundance across all samples, and the remaining taxa are labeled as "other." **B.** Taxa bar plot of 26 sample pairs from patients with UC, showing samples from areas without inflammation (UC-F) and with inflammation (UC-T).
