# Supplementary material for: Live Malassezia strains from the mucosa of patients with ulcerative colitis: pathogenic potential and environmental adaptations
Source: mBio. 2025 Jun 13;16(7):e01400-25. doi: 10.1128/mbio.01400-25 (PMC12239588; doi:10.1128/mbio.01400-25)
Supplement: Figure S3 — The results of the rank test. [file mbio.01400-25-s0003.pdf]

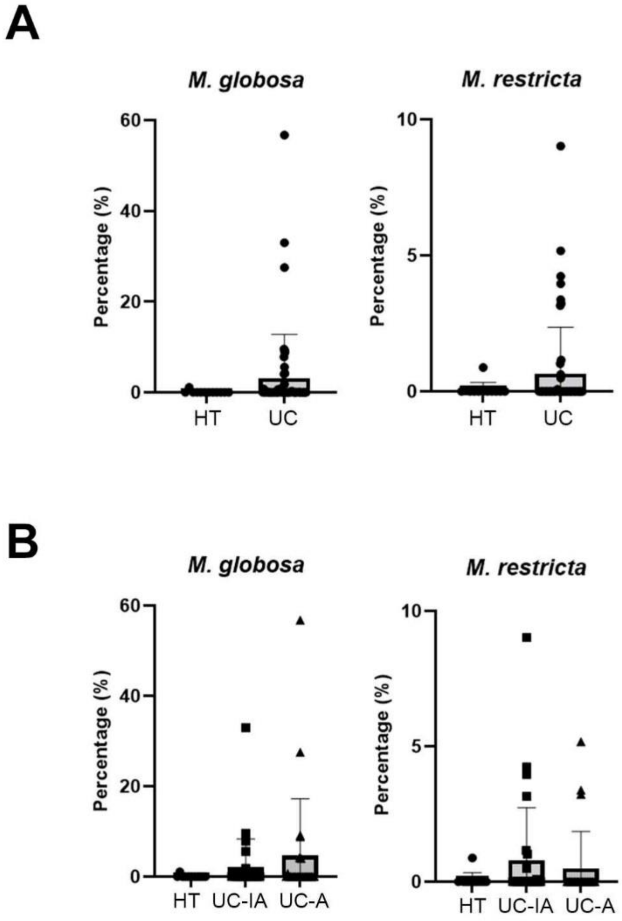

Fig. S3. A. The rank test revealed that more *M. globosa* and *M. restricta* reads were present in UC samples than in healthy control samples (HT); however, it was insignificant. **B.** The rank test revealed no significant difference in the abundance of *M. globosa* and *M. restricta* between the gut mucosal surfaces without (UC-IA) and with (UC-A) inflammation, respectively, in the same patient with UC.
