# Supplementary material for: Live Malassezia strains from the mucosa of patients with ulcerative colitis: pathogenic potential and environmental adaptations
Source: mBio. 2025 Jun 13;16(7):e01400-25. doi: 10.1128/mbio.01400-25 (PMC12239588; doi:10.1128/mbio.01400-25)
Supplement: Figure S4 — Venn diagrams to determine common genes showing differential regulation between isolates. [file mbio.01400-25-s0004.pdf]

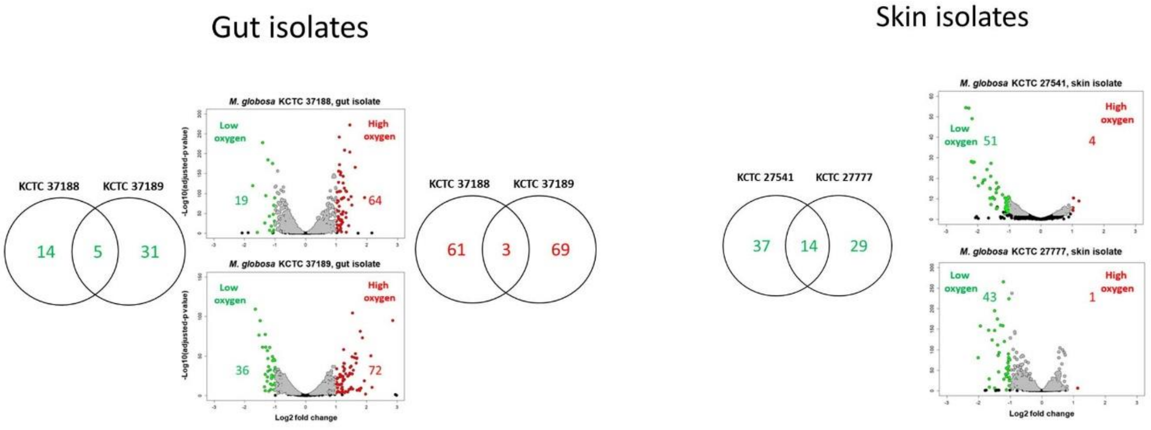

Fig. S4. Venn Diagrams to determine common genes showing differential regulation between isolates. 5 and 3 genes were identified as common genes between the gut isolates (KCTC 37188 vs. KCTC 37189) under low- and high-oxygen conditions, respectively. A total of 14 genes were identified as common genes between the skin isolates (KCTC 27541 vs. KCTC 27777), but only under low-oxygen conditions.
