# Supplementary material for: Live Malassezia strains from the mucosa of patients with ulcerative colitis: pathogenic potential and environmental adaptations
Source: mBio. 2025 Jun 13;16(7):e01400-25. doi: 10.1128/mbio.01400-25 (PMC12239588; doi:10.1128/mbio.01400-25)
Supplement: Figure S5 — Growth comparison assay. [file mbio.01400-25-s0005.pdf]

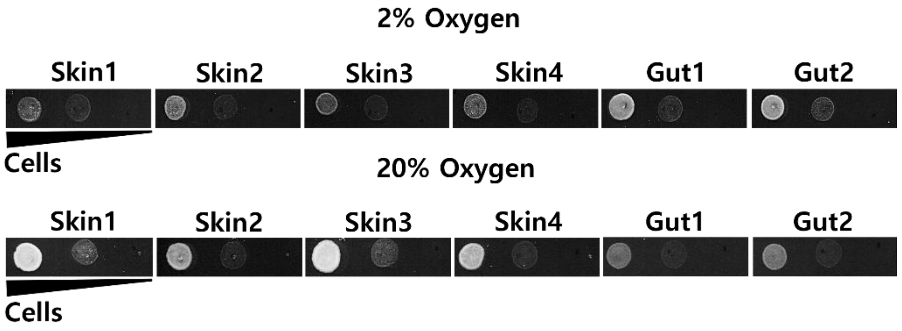

Fig. S5. Growth comparison assay. The growth of the gut and the skin isolates in the presence of two different oxygen concentrations. Serially diluted cells of each isolate (10^8, 10^7, and 10^6 cells) were spotted on mDixon media, incubated at 34C for two days in the presence of 2 or 20 % oxygen, and photographed. Skin 1: KCTC27541; Skin 2: KCTC27777; Skin 3: KCTC27523; Skin 4: KCTC27816; Gut 1: KCTC37188; Gut 2: KCTC37189.
