# Supplementary material for: Live Malassezia strains from the mucosa of patients with ulcerative colitis: pathogenic potential and environmental adaptations
Source: mBio. 2025 Jun 13;16(7):e01400-25. doi: 10.1128/mbio.01400-25 (PMC12239588; doi:10.1128/mbio.01400-25)
Supplement: Figure S6 — In vivo competition assay. [file mbio.01400-25-s0006.pdf]

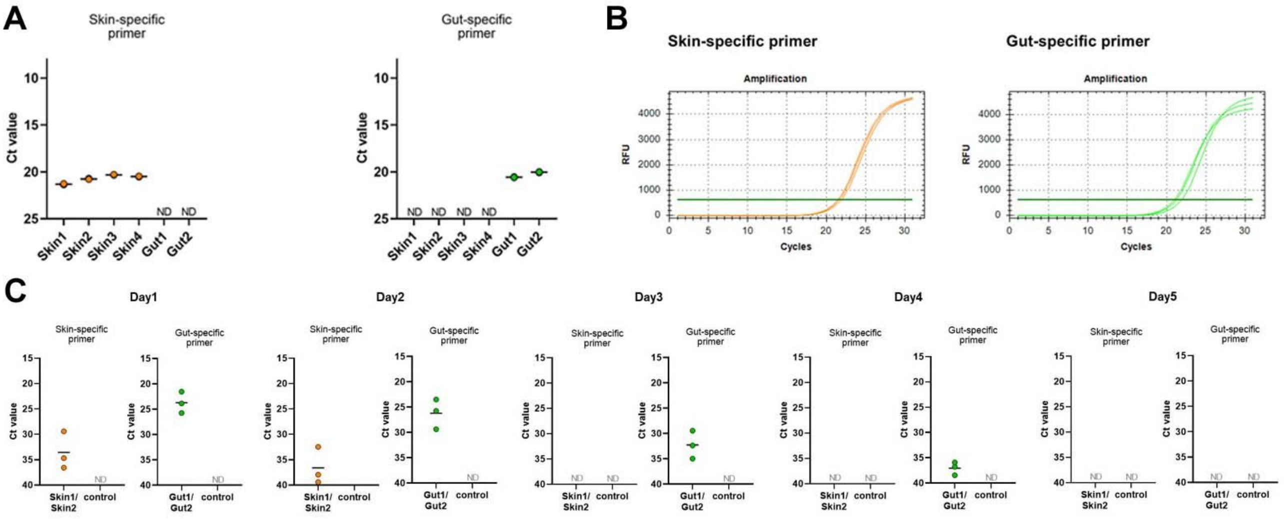

Fig. S6. A. The specificity of the primers was validated by qPCR using genomic DNA from each strain, including all four skin isolates used in the current study, before applying them to the competition assay. The results confirmed that the primers are highly specific to the gut and the skin isolates and properly distinguish between the strains. **B**. DNA was also extracted from the same fungal inoculum and subjected to qPCR using skin isolate- or gut isolate-specific primers. Similar Ct values, an average of 21.66 and 21.39 from three replicate analyses using the skin isolate- and the gut isolate-specific primers, respectively, indicate that equal amounts of the skin and the gut isolates were added to the inoculum. **C**. In vivo competition assay in DSS-induced colitis mice. The same number, 1×10^7 cells, of skin 1, skin 2, gut 1, and gut 2 strains were mixed and orally gavaged once into the mice with DSS-induced colitis. The feces of the mice were harvested daily. Presence of each strain within feces was evaluated by qPCR using DNA extracted from fecal samples and the skin isolates- or the gut isolates-specific primers. Orange circles represent skin isolates (Skin1 and Skin2), and green circles represent gut isolates (Gut1 and Gut2). The y-axis shows Ct values, with lower values indicating higher amounts of DNA. Control represents uninfected mice. ND indicates "Not Detected." Data points represent mean values from three independent experiments. Note that the skin isolates were detected until day 2 (orange dots) while the gut isolates were detected until day 4 (green dots). The gut isolates were no longer detected after day 5. The horizontal bar represents the median of the observed competitive index values obtained from three independent measurements using three mice.
