# Supplementary material for: Live Malassezia strains from the mucosa of patients with ulcerative colitis: pathogenic potential and environmental adaptations
Source: mBio. 2025 Jun 13;16(7):e01400-25. doi: 10.1128/mbio.01400-25 (PMC12239588; doi:10.1128/mbio.01400-25)
Supplement: Figure S7 — Relative quantification of M. globosa DNA in G. mellonella larvae. [file mbio.01400-25-s0007.pdf]

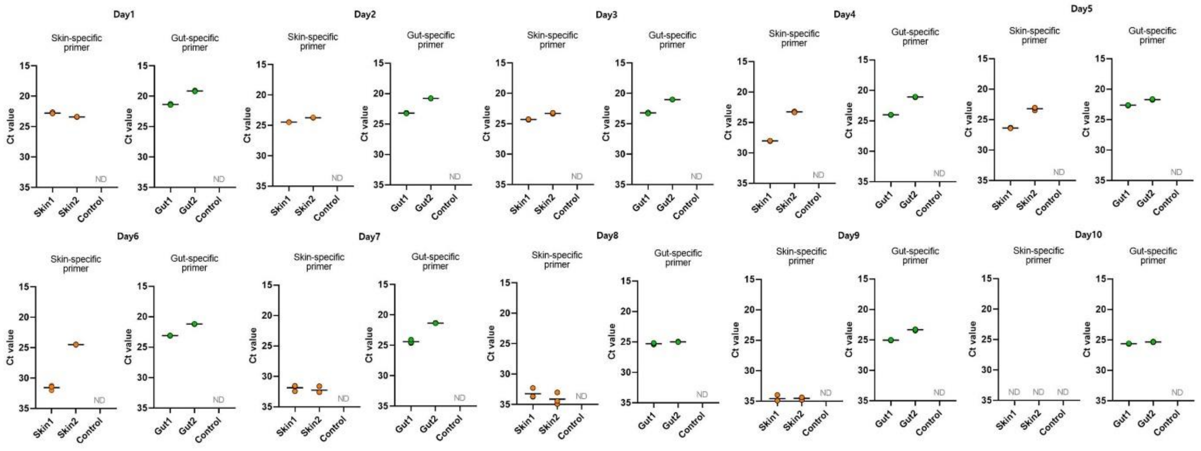

Fig. S7. Relative quantification of *M. globosa* DNA in *G. mellonella* larvae over time using strain-specific primers. The persistence of M. globosa skin and gut isolates in *G. mellonella* larvae was monitored for 10 days using qPCR with strain-specific primers. DNA was extracted from homogenized larvae daily and analyzed using both skin isolates-specific and gut isolates-specific primers. Orange circles represent skin isolates (Skin1 and Skin2), and green circles represent gut isolates (Gut1 and Gut2). The y-axis shows Ct values, with lower values indicating higher amounts of DNA. Control represents uninfected larvae. ND indicates "Not Detected." Data points represent mean values from three independent experiments. The gut isolates maintained detectable levels of DNA throughout the 10-day period, while DNA levels of the skin isolates decreased after day 7, suggesting better persistence of gut isolates in the larval environment.
