# Supplementary material for: Live Malassezia strains from the mucosa of patients with ulcerative colitis: pathogenic potential and environmental adaptations
Source: mBio. 2025 Jun 13;16(7):e01400-25. doi: 10.1128/mbio.01400-25 (PMC12239588; doi:10.1128/mbio.01400-25)
Supplement: Table S1 — List of patients (healthy controls and patients with UC): age, sex, disease information, and treatment data. [file mbio.01400-25-s0008.pdf]

Table S1. List of patients included in the current study.

Sample ID	Gender/Age	HT/PT	Sampling site	Inflammation (-/+)
13	M/40	Ulcerative colitis	Sigmoid colon	+
14	M/40	Ulcerative colitis	Sigmoid colon	-
*19	F/62	Ulcerative colitis	Descending colon	-
20	M/47	Ulcerative colitis	Descending colon	-
21	M/47	Ulcerative colitis	Sigmoid colon	-
*22	F/71	Ulcerative colitis	Descending colon	-
25	M/41	Ulcerative colitis	Transverse colon	-
26	M/41	Ulcerative colitis	Sigmoid colon	+
27	M/66	Ulcerative colitis	Sigmoid colon	+
28	M/66	Ulcerative colitis	Rectum	-
31	F/37	Ulcerative colitis	Rectum	+
32	F/37	Ulcerative colitis	Descending colon	-
35	F/52	Ulcerative colitis	Sigmoid colon	+
36	F/52	Ulcerative colitis	Descending colon	-
38	F/20	Ulcerative colitis	Sigmoid colon	+
39	F/20	Ulcerative colitis	Transverse colon	-
42	M/62	Ulcerative colitis	Descending colon	+
43	M/62	Ulcerative colitis	Sigmoid colon	-
50	M/63	Ulcerative colitis	Sigmoid colon	+
51	M/63	Ulcerative colitis	Transverse colon	-
54	M/28	Ulcerative colitis	Descending colon	+
55	M/28	Ulcerative colitis	Rectum	-
69	F/33	Ulcerative colitis	Transverse colon	-
70	F/33	Ulcerative colitis	Descending colon	+
79	M67	Ulcerative colitis	Sigmoid colon	+
80	M67	Ulcerative colitis	Descending colon	-
84	M/30	Ulcerative colitis	Sigmoid colon	+
85	M/30	Ulcerative colitis	Descending colon	-
90	M/74	Ulcerative colitis	Sigmoid colon	+
91	M/74	Ulcerative colitis	Transverse colon	-
98	M/57	Ulcerative colitis	Descending colon	-
99	M/57	Ulcerative colitis	Appendiceal orifice	+
104	M/29	Ulcerative colitis	Rectum	+
105	M/29	Ulcerative colitis	Transverse colon	-
106	F/33	Ulcerative colitis	Ascending colon	+
107	F/33	Ulcerative colitis	Descending colon	-
108	M/71	Ulcerative colitis	Rectum	+
109	M/71	Ulcerative colitis	Sigmoid colon	-
110	M/24	Ulcerative colitis	Ascending colon	-
111	M/24	Ulcerative colitis	Sigmoid colon	+
114	F/32	Ulcerative colitis	Sigmoid colon	
115	F/32	Ulcerative colitis	Descending colon	+
116	F/59	Ulcerative colitis	Sigmoid colon	+
117	F/59	Ulcerative colitis	Sigmoid colon	-
118	F/79	Ulcerative colitis	Sigmoid colon	+
119	F/79	Ulcerative colitis	Rectum	-
120	M/71	Ulcerative colitis	Rectum	-
121	M/71	Ulcerative colitis	Sigmoid colon	+
122	M/45	Ulcerative colitis	Sigmoid colon	+
123	M/45	Ulcerative colitis	Transverse colon	-
124	F/50	Ulcerative colitis	Rectum	+
125	F/50	Ulcerative colitis	Transverse colon	-
126	F/35	Ulcerative colitis	Descending colon	+
127	F/35	Ulcerative colitis	Sigmoid colon	-
131	M/28	Ulcerative colitis	Ascending colon	-
132	M/28	Ulcerative colitis	Descending colon	+

* All samples were paired and collected from the site indicated with and without inflammation in the same patient except sample 19 and 22.

Sample ID	Gender/Age	HT/PT	Sampling site	Inflammation (-/+)
29	M/74	HT	Sigmoid colon	-
40	F/67	HT	Sigmoid colon	-
41	M/74	HT	Descending colon	-
44	F/62	HT	Sigmoid colon	-
45	M/42	HT	Sigmoid colon	-
49	F/56	HT	Descending colon	-
72	F/57	HT	Descending colon	-
73	F/52	HT	Descending colon	-
128	F/53	HT	Ascending colon	-
129	M/73	HT	Ascending colon	-
130	F/65	HT	Descending colon	-
