# Supplementary material for: Live Malassezia strains from the mucosa of patients with ulcerative colitis: pathogenic potential and environmental adaptations
Source: mBio. 2025 Jun 13;16(7):e01400-25. doi: 10.1128/mbio.01400-25 (PMC12239588; doi:10.1128/mbio.01400-25)
Supplement: Table S2 — Live fungal strains obtained in this study. [file mbio.01400-25-s0009.pdf]

Table S2. Live fungal strains obtained in the current study.

Phylum	PT
**Ascomycota**	*Candida albicans*<br>*Candida glabrata*<br>*Candida tropicalis*<br>*Candida orthopsilosis*<br>*Candida bracarensis*<br>*Candida parapsilosis*<br>*Cyberlindnera fabianii*<br>*Pichia kluyveri*<br>*Pichia guilliermondii*<br>*Aspergillus niger*<br>*Aspergillus unguis*<br>*Meyerozyma carpophila*<br>*Meyerozyma guilliermondii*<br>*Saccharomyces cerevisiae*<br>*Wickerhamomyces anomalus*<br>*Kazachstania servazzii*<br>*Kazachstania aerobia*<br>*Coniochaeta hoffmannii*<br>*Clavispora lusitaniae*
**Basidiomycota**	*Malassezia furfur*<br>*Malassezia obtusa*<br>*Malassezia globosa*<br>*Daedaleopsis confragosa*<br>*Trametes versicolor*<br>*Rhodotorula mucilaginosa*<br>*Cryptococcus diffluens*<br>*Rhodosporidiobolus sp.*
**Mucoromycota**	*Rhizomucor pusillus*

Phylum	HT
**Ascomycota**	*Candida albicans*<br>*Candida glabrata*<br>*Candida tropicalis*<br>*Candida orthopsilosis*<br>*Candida parapsilosis*<br>*Cyberlinera fabianii*
**Basidiomycota**	*Malassezia sympodialis*

PT: Isolates from ulcerative patients

HT: Isolates from healthy individuals
